# Isotemporal Substitution as the Gold Standard Model for Physical Activity Epidemiology: Why It Is the Most Appropriate for Activity Time Research

**DOI:** 10.3390/ijerph16050797

**Published:** 2019-03-05

**Authors:** Rania A. Mekary, Eric L. Ding

**Affiliations:** 1 Department of Nutrition, Harvard T.H. Chan School of Public Health, Boston, MA 02115, USA; eding@post.harvard.edu; 2 Department of Pharmaceutical Business and Administrative Sciences, School of Pharmacy, MCPHS University, 179 Longwood Avenue, Boston, MA 02115, USA; 3 Department of Neurosurgery, Computational Neuroscience Outcomes Center, Brigham and Women’s Hospital, Harvard Medical School, 15 Francis Street, Boston, MA 02115, USA

Isotemporal substitution model was first developed in 2009 [1] by our research team, whose members also once developed the isocaloric substitution model, widely-regarded as the gold standard method for modeling calorie-containing foods in nutritional epidemiology [2,3,4]. The ISM was developed because epidemiology research in daily activity time, up until recently, did not incorporate that daily time of 24 h a day is finite.

Thus, as the original creators of the isotemporal substitution model for physical activity time allocation analysis [1], we read with interest the paper by Biddle and colleagues [5] which described the association between behavior reallocations and risk of metabolic health in a UK cohort. The authors reported the results using the compositional data analysis developed by Chastin et al. in 2015 [6], highlighting the differences between this model and what they called the “traditional” isotemporal substitution model (ISM) developed by Mekary et al. in 2009 [1]. While we commend the authors for their attempt to compare the two methods, we disagree with the explanations given to justify the superiority of the compositional data analysis over the ISM. In fact, compositional analysis is simply a derivative form of ISM that has been already shown in nutritional epidemiology to be a similar model mathematically.

Frist, Biddle and colleagues [5] mentioned that while the ISM used absolute values in physical activity, the compositional data analysis used relative values. Interestingly, we have previously discussed the feasibility of using relative values (as in percentages) rather than absolute values in the ISM—we called it the “density model”, in reference to the “multivariate nutrient density model” developed by Willett and Stampfer (1986) [2,3,4] that is being used in nutrition epidemiology. We recommended against the use of the density model in physical activity epidemiology because unlike nutrition intake, which could be given in relative amounts or percentages, physical activity guidelines are given in absolute amounts [7] (e.g., 30 min/day of weight training) instead of relative amounts (e.g., 5% of your waking time spent in weight training). Similarly, a certain percentage of total discretionary activity time could be very heterogeneous among individuals, which makes it hard to interpret and establish physical activity guidelines because different individuals often have widely different total discretionary time available for physical activities. Thus, the same 30 min of running could represent a very different percentage of total activity time. Furthermore, unlike nutrition, almost all physical activity guidelines are given in terms of absolute values and not relative values. These values could vary by age group [7] or by desired outcome (weight loss maintenance [8], weight gain prevention [9], waist circumference change [10], cardiovascular health [11], among others); yet, they do not vary for every individual. In our opinion, this makes the density model, also called compositional data analysis by Chastin et al. (2015) [6], inappropriate to use in physical activity epidemiology. It also lends itself to potentially very inaccurate interpretations by different individuals of varying discretionary activity time.

Second, Biddle and colleagues [5] compared their findings using both models and noticed that while the ISM led to symmetric results for the reallocation of behaviors, compositional data analysis led to asymmetric results, which in their opinion was a more accurate estimation of the effect size. For instance, they argued that substituting 30 min/day of moderate to vigorous physical activity for an equal time of sedentary behavior might not necessarily lead to the inverse effect estimate when this substitution was reversed. In our opinion, this depends on the model used. In fact, if data were normally distributed—according to the central limit theorem—and a linear regression model was used to model these associations, symmetry in the results would be expected if substitutions in activities were reversed as associations were estimated in an additive way. However, if the data were not normal or if there were more outliers than expected, the natural log of the outcome would then be modeled and associations would be estimated in a multiplicative way. The symmetry would then be lost in this model. Of note, we showed similar asymmetry in Mekary et al. (2013) [12] when a binary outcome of depression was modeled as an outcome via the Cox proportional hazard model to assess the relationship between activity and clinical depression risk. Similarly, in the paper by Biddle and colleagues [5], the authors used isometric log-ratios as predictor variables. In the original paper by Chastin et al. (2015) [6], the authors log-transformed the non-normally distributed outcomes. This natural log transformation took away the symmetry, as when the data were transformed back to the original scale, the reversal of substitutions of behaviors would no longer lead to symmetrical results. Hence, we disagree with the claim the authors that the ISM led to inaccurate results, which were symmetrical in this case. The ISM model could lead to symmetrical or asymmetrical results based on the scale used, as previously shown [1,12]; thus, symmetry is not a metric of any given model’s superiority per se.

Third, Biddle and colleagues [5] compared their findings obtained using the compositional data analysis with the ISM model and noted that results were not materially different. Interestingly, the same conclusions were drawn using either model. Notably, the results from ISM were more appropriate, intuitive, and easy to understand, while the results from the compositional data analysis were hard to articulate. The authors, nevertheless, interpreted their findings using the same language used to interpret the ISM. This simply confirms what is already known from nutritional epidemiology’s decades of research of isocaloric substitution that have compared different substitution approaches [13], where substitution models with absolute macronutrient intakes have shown similar if not the same results as energy density (i.e., calorie percentage) models. Thus, their study simply reconfirms what is already known mathematically, all while confusing the literature with a seemingly different analysis that is more difficult to interpret for physical activity.

Taken together, we stand by our original ISM and the accuracy and superior interpretation of the emanating results for physical activity. Other authors have even called the original ISM model a ‘seminal work’ in a recent systematic review of 56 ISM papers worldwide [14]. Moreover, the statistical properties of the ISM encompass those of derivative models, such as compositional analysis, which is merely ISM in another equivalent form. Altogether, we believe that the ISM in absolute units is the appropriate model to use in the arena of physical activity epidemiology, given that physical activity guidelines are provided and conveyed in absolute values rather than relative values.

## Abbreviation

ISM	isotemporal substitution model
